# Ileal Perforation and Enteric Fever: Implications for Burden of Disease Estimation

**DOI:** 10.1093/infdis/jiab258

**Published:** 2021-11-23

**Authors:** Swathi Krishna Njarekkattuvalappil, Maria Thomas, Arti Kapil, Karnika Saigal, Pallab Ray, Shalini Anandan, Savitha Nagaraj, Jayanthi Shastri, Sulochana Putli bai Perumal, Dasaratha Ramaiah Jinka, Shajin Thankaraj, Vijayanand Ismavel, Pradeep Zachariah, Ashita Singh, Madhu Gupta, Sheena Evelyn Ebenezer, Mathew Santosh Thomas, Dhruva Ghosh, Kamal Kataria, Mamta Senger, Sundaram Balasubramanian, Gagandeep Kang, Jacob John

**Affiliations:** 1 Christian Medical College, Vellore,India; 2 Christian Medical College and Hospital, Ludhiana,India; 3 All India Institute of Medical Sciences, New Delhi,India; 4 Chacha Nehru Bal Chikitsalaya, New Delhi,India; 5 Post Graduate Institute of Medical Education and Research, Chandigarh,India; 6 St John’s Medical College, Bangalore,India; 7 Topiwala National Medical College and BYL Nair Charitable Hospital, Mumbai,India; 8 Kanchi Kamakoti Childs Trust Hospital, Chennai,India; 9 Rural Development Trust Hospital, Bathalapalli, Andhra Pradesh,India; 10 Makunda Christian Leprosy and General Hospital, Bazaricherra, Assam,India; 11 Lady Willingdon Hospital, Manali, Himachal Pradesh,India; 12 Chinchpada Christian Hospital, Maharashtra,India; 13 The Duncan Hospital, Raxaul, Bihar,India

**Keywords:** burden estimates, case fatality rate, enteric fever, Meckel diverticulum, nontraumatic ileal perforation, surveillance, tuberculosis

## Abstract

**Background:**

Ileal perforation occurs in about 1% of enteric fevers as a complication, with a case fatality risk (CFR) of 20%–30% in the early 1990s that decreased to 15.4% in 2011 in South East Asia. We report nontraumatic ileal perforations and its associated CFR from a 2-year prospective enteric fever surveillance across India.

**Methods:**

The Surveillance for Enteric Fever in India (SEFI) project established a multitiered surveillance system for enteric fever between December 2017 and March 2020. Nontraumatic ileal perforations were surveilled at 8 tertiary care and 6 secondary care hospitals and classified according to etiology.

**Results:**

Of the 158 nontraumatic ileal perforation cases identified,126 were consented and enrolled. Enteric fever (34.7%), tuberculosis (19.0%), malignancy (5.8%), and perforation of Meckel diverticulum (4.9%) were the common etiology. In those with enteric fever ileal perforation, the CFR was 7.1%.

**Conclusions:**

Enteric fever remains the most common cause of nontraumatic ileal perforation in India, followed by tuberculosis. Better modalities of establishing etiology are required to classify the illness, and frame management guidelines and preventive measures. CFR data are critical for comprehensive disease burden estimation and policymaking.

Enteric fever includes typhoid fever caused by *Salmonella* Typhi and paratyphoid fever caused by *Salmonella* Paratyphi A, B, and C. Historically, enteric fever was prevalent all over the world. At the turn of the 20th century, the emergence of safe, reticulated water systems, improved sanitation, and better food hygiene resulted in enteric fever and its complications being eliminated in the industrialized world, but the disease persists in regions of poor sanitation, crowding, and poverty [1–3]. Ileal perforation is a potentially fatal complication of enteric fever, which usually occurs by the third week of the disease when it is treated inadequately or left untreated. In general, intestinal hemorrhage and perforation tend to occur in the terminal ileum secondary to necrosis of Peyer patches [4]. Mortality from ileal perforation due to typhoid fever has declined worldwide with the use of antibiotics, surgical care, and supportive therapy [5].

Limited data from hospital-based studies from Southeast Asia indicate a 1% prevalence of ileal perforation as a complication among blood culture-confirmed enteric fever [6, 7]. The case fatality rate (CFR) of typhoid intestinal perforation in Asia and Africa declined from 20%–30% in the early 1990s to 15.4% in 2011 [8]. Timely diagnosis and surgical intervention remain a challenge in low- and middle-income countries of the world. A constraint in formulating treatment guidelines and recommendations for enteric fever ileal perforations is the lack of adequate and reliable prospective surveillance data [9].

In enteric fever, the ingested organism enters the small intestine and, via the M cells of the Peyer patches, migrates into the mesenteric lymph nodes followed by transient primary bacteremia, localization and multiplication in multiple organs, and secondary bacteremia. By the third week of illness, ulcers may form due to necrosis in the Peyer patches on the antimesenteric border of the intestine, and these may result in perforation. The usual site of perforation is the terminal ileum and 75% of cases comprise single perforations, occurring after 20 days of illness [10, 11]. The diseased gut is characterized by diffuse nonspecific enterocolitis with hypertrophy, necrosis, and ulceration of intestinal and mesenteric lymphatic tissue. Acute and chronic inflammatory cells are involved with a predominance of CD68^+^ leucocytes (macrophages) and CD3^+^ T lymphocytes at the perforation sites [1, 12–14]. Limited data (Table 1) are available on perforations from southern Asia. The leading infectious causes are enteric fever and tuberculosis, and hence this condition is mostly preventable. While an effective vaccine is available to prevent enteric fever, lack of robust data on disease burden and its complications delays its programmatic use. Limited and less sensitive diagnostic modalities for enteric fever also add to the underestimation of disease burden.

**Table 1.** Existing Literature on Etiology of Ileal Perforation

Study	Leading Infectious Causes for Perforation, No. of Cases (%)	Age Group Studied and Study Duration
Poornima et al 2017 Bengaluru, India [15]	59 (82.81) enteric fever	21–67 y
	5 (7.81) tuberculosis	June 2011 to May 2015
	6 (9.38) nonspecific	
Singh et al 2014 Pune, India [16]	4 (57) enteric fever	30–50 y
	3 (43) nonspecific	2009 to 2010
Wani et al 2006 Kashmir, India [17]	49 (62) enteric fever	All age groups
	3 (4) tuberculosis	Mean age = 34.62 y (SD 14.16)
	21 (26) nonspecific	
		January 1999 to July 2005
Verma et al 2015 Rohtak, India [18]	10 (24.4) enteric fever	All age groups
	8 (19.5) tuberculosis	Mean age = 38.31 y (SD 18.99)
	23 (56) nonspecific	August 2011 to December 2013
Khalid et al 2014 Lahore, Pakistan [19]	82 (65.6) enteric fever	All age groups
	38 (30.4) tuberculosis	Mean age = 22.96 y (SD 4.8)
	5 (4) nonspecific	January 2014 to November 2014
Anam et al 2018 Faisalabad, Pakistan [20]	14 (11.8) typhoid	0–90 y
	3 (2.5) tuberculosis	June 2017 to November 2017
	66 (55.5) nonspecific	

To quantify the complications caused due to severe enteric fever, a substudy to capture nontraumatic intestinal perforations was embedded within the multisite, multitier Surveillance for Enteric Fever in India (SEFI) project with active case recruitment and follow-up in the collaborating centers.

## METHODS

### Study Setting

Eight tertiary care hospitals and 6 secondary care hospitals, in the public and private sectors across India, were part of the surveillance network under SEFI (Supplementary Table 1).

### Study Period

A surveillance for nontraumatic ileal perforations was established in parallel to the facility-based and laboratory surveillance for enteric fever between December 2017 and March 2020.

### Inclusion Criteria, Enrolment, and Follow-up

All cases of nontraumatic ileal perforations, irrespective of etiology, undergoing surgical intervention from the pediatric and adult surgical departments at the study sites were invited to participate in the surveillance. Structured questionnaires were used to collect data on sociodemographic characteristics, the severity of disease, and the cost of illness. The study participants were recontacted on day 28 postdiagnosis and the outcome and cost of illness information was collected.

### Etiology of Nontraumatic Ileal Perforations

SKN and JJ independently assigned causality based on available clinical data, laboratory evidence including blood culture (positive for enteric), Widal test (>1:160 dilution for *Salmonella* O antigen), and surgical notes or histopathological evidence, as in Table 2. All data were collected on structured questionnaires and entered into a centrally managed web-based electronic data management system.

**Table 2.** Schema for Classification of Ileal Perforation Cases into Categories

Category	Clinical Evidence	Laboratory Evidence	Surgical or Histopathological Evidence
Confirmed EF	Pointing to EF	Blood culture or Widal positive within the study facility or done outside with documented reports	±
Probable EF	Pointing to EF	Blood culture or Widal positive by patient’s history but no report available	±
Possible EF	Inconclusive	Inconclusive	±
Not EF	No evidence of EF, other specific diagnosis is assigned by physician		

Abbreviation: EF, enteric fever.

### Ethical Considerations

The protocol of the study was approved by the ethics committees of the Christian Medical College Vellore and those of the participating sites. Written informed consent was obtained from all participants or their legally authorized representatives.

## RESULTS

The surveillance received reports of 158 nontraumatic ileal perforation cases from the 14 study sites. Of these, 12 patients could not be contacted for consent, 20 patients refused consent, 4 had history and clinical findings that were not consistent with a diagnosis of nontraumatic ileal perforation, while 1 recruited patient had inadequate data for categorization as nontraumatic ileal perforation. Therefore, 126 patients were enrolled, and 121 cases were included in the analysis.

Of the 121 analyzed episodes, 34 (28.09%) were younger than 15 years (Table 3). The mean age was 26.4 years, and 78 (64.5%) were male. Sixteen reported an episode of enteric fever in the previous year including 4 that were identified by blood culture.

**Table 3.** Demographic Features of Enrolled Ileal Perforation Cases (n = 121)

Characteristic	Value
Age	
Pediatric (<15 y)	34 (28.10)
Adult	87 (71.9)
Median age	25 y (0–83)
Mean age (SD)	26.4 y (20.1)
Sex	
Male	78 (64.5)
Female	43 (35.5)
Previous history of enteric fever	
Yes, confirmed by blood culture	4 (3.3)
Yes, but unconfirmed by blood culture	12 (9.92)
No	96 (79.34)
Not known	9 (7.44)

Data are No. (%) except where indicated.

In 19 (15.7%) cases there was a history of fever, abdominal pain, and vomiting and either a blood culture that grew *Salmonella* Typhi/Paratyphi or a Widal test that was positive. These were classified as enteric fever (Table 4). Seven (5.8%) patients had clinical features indicative of enteric fever with an unverified history of blood culture or Widal positivity and these were classified as probable enteric fever perforations; 16 (13.2%) patients had clinical features and laboratory evidence supporting generalized sepsis but not enough information to rule enteric fever out and were classified as possible enteric fever perforations; 65 (53.7%) patients had other specific diagnoses assigned and these were classified as the not enteric fever perforations; and 14 (11.6%) cases were unclassified due to inadequate clinical data. Histopathology reports were available for 58 patients. Of the 65 nonenteric fever-related perforations, 23 (35.3%) were due to tuberculosis, 7 (10.8%) due to malignancy, 10 (15.3%) due to congenital causes, and 3 (4.6%) due to inflammatory bowel disease.

**Table 4.** Classification of Nontraumatic Ileal Perforation Cases Enrolled (n = 121)

Category	No. (%)
Confirmed EF	19 (15.7)
Probable EF	7 (5.8)
Possible EF	16 (13.22)
Not EF	65 (53.7)
Unclassified (inadequate data)	14 (11.6)

Abbreviation: EF, enteric fever.

All 121 cases underwent surgical repair. Most of the perforations were in the distal/terminal ileum (66.1%), and the distribution of single versus multiple perforations was similar (52.1% vs 47.9%). The mean duration of hospitalization was 19.2 days and 71.9% of the cases recovered without complications. The documented fatality rate was 22.3% while 4.1% left against medical advice (Table 5).

**Table 5.** Characteristics of Ileal Perforations (n = 121)

Characteristics	Confirmed EF (n = 19)	Probable EF (n = 7)	Possible EF (n = 16)	Not EF (n = 65)	Unclassified (n = 14)
Number of perforation(s)					
Single	11	3	8	31	10
Multiple	8	4	8	34	4
Site of perforation(s)					
Proximal ileum	5	1	5	26	4
Distal ileum	14	6	11	39	10
Type of surgical repair					
Primary closure, single/double layered	8	2	3	4	2
Primary closure with omental patch	0	0	0	1	0
Resection and anastomosis	2	1	2	16	2
Ileostomy	7	3	4	17	4
Primary closure with ileotransverse colostomy	0	0	0	1	0
Others	2	1	7	26	6
Duration of hospital stay, d, median (range)	16 (6–32)	13 (1–44)	16.5 (6–94)	16 (3–56)	15 (6–52)
Duration of hospital stay, d, mean (SD)	17.4 (7.7)	18.5 (14.5)	22.8 (21.7)	18.7 (11.9)	17.9 (11.5)
Outcome of the episode at discharge					
Recovered without complications	18	6	12	46	5
Recovered with complications	0	0	2	0	0
Referred to other hospital	0	0	0	0	0
Death	0	1	2	18	6
Left against medical advice	1	0	0	1	3

Data are No. of cases except where indicted.

Abbreviation: EF, enteric fever.

Of the enrolled cases, 27 (22.3%) died, including 3 among 42 who could have had enteric fever-related perforation. The CFR for enteric fever-related nontraumatic ileal perforation was 7.1% (95% confidence interval [CI], 2.45%–19.01%). The nontraumatic ileal perforations that were considered to be due to tuberculosis (6/23) had a case fatality of 26% (95% CI, 12.5%–46.5%) and about half of the tuberculosis perforation had multiple ulcers in ileocecal region (11/23, 47.8%). A description of the clinical course for a few representative cases is provided in Box 1.

Box 1. **Case Summaries**

**Case-1**

A 55-year-old man with complaints of fever, abdominal pain, constipation, and vomiting for 5 days with a positive Widal test conducted at an outside laboratory. There was no previous history of typhoid fever. The patient underwent a surgical closure of single, distal ileal perforation and an ileostomy was done. Postoperatively intravenous metronidazole, cefotaxime, ceftriaxone, and amikacin were administered. However, the patient’s condition worsened after a few hours, progressing to septic shock with multiorgan dysfunction syndrome, and finally death on the same day.

**Case-2**

A 10-year-old boy from an upper middle-class family in Chennai, with fever, abdominal pain, vomiting, and anorexia for 3 days who was being treated with antibiotics by a private practitioner. At the hospital, he underwent an exploratory laparotomy in which a single terminal ileal perforation was detected and primary closure was done. Blood culture revealed no growth. Postoperatively, intravenous piperacillin and tazobactam was given and the child recovered without further complications. He was discharged on day 6 postadmission. This child presented to outpatient department 2 months later and a blood culture done at that time was positive for *Salmonella* Typhi. However, the patient was not admitted at the study hospital and no further data are available.

**Case-3**

A 17-year-old boy who was a factory worker residing in Ludhiana, with fever, abdominal pain, and breathlessness for the past 2 weeks and showing features of hemodynamic shock was admitted in the hospital and diagnosed to have multiple proximal ileal perforations for which he underwent primary closure. Blood culture grew *Salmonella* Typhi and he was treated with intravenous piperacillin plus tazobactam for 6 days, vancomycin for 8 days, amikacin for 13 days, and ceftriaxone for 12 days. The blood culture isolate was sensitive to azithromycin, ceftriaxone, chloramphenicol, cipro/pefloxacin, and cotrimoxazole. The tissue biopsy was, however, negative for histopathological features as well as *Salmonella* DNA by polymerase chain reaction. He had a hospital stay of 28 days including 3 days in the intensive care unit and recovered without any further complications.

## Discussion

From the SEFI surveillance, the prevalence of ileal perforation as a complication among blood culture-positive typhoid fever patients was 0.32% (3/960). A recent systematic review of complications of typhoid fever by Cruz Espinoza et al reported prevalence of intestinal perforation as a complication of typhoid as 1.1% (0.4%–1.8%) [21]. This study, embedded in the SEFI surveillance, enrolled all the nontraumatic ileal perforation cases from the adult and pediatric surgical departments of the study hospitals, irrespective of the cause of perforation. We identified 121cases with ileal perforations in 14 Indian hospitals, of which 19 were considered to be due to enteric fever, 7 were probable, and 16 were possible, indicating that complications of enteric fever continue to occur despite the availability and widespread indiscriminate use of effective antibiotics.

The major etiologies of nontraumatic ileal perforations here were enteric fever (34.7%), tuberculosis (19%), malignancy (5.8%), and perforation of Meckel diverticulum (4.9%). These findings are in concordance with those in various studies in developing countries of Asia and Africa [15–22] wherein the observational studies showed predominance of enteric fever as the leading cause for nontraumatic ileal perforation.

In the Surveillance for Enteric Fever in Asia Project (SEAP) conducted in Pakistan, Nepal, and Bangladesh from 2016 to 2019, large number of ileal perforations were reported from Pakistan, which also coincided with the outbreak of XDR strains of *Salmonella* in the country [23]. Although our study in India did not reveal any drug-resistant strains among the perforation cases, it is a warning signal to act upon the prevailing problem prophylactically. Ileal perforation as a complication of untreated or inadequately treated enteric fever, usually occurring by the third week of illness [24]. Laboratory confirmation is generally difficult at this stage because blood and bone marrow cultures often yield no growth [25, 26]. Widal tests are used widely but are not recommended in the absence of other confirmatory evidence [27]. Of the 42 possible cases of enteric fever ileal perforations, laboratory confirmation in terms of a blood culture or Widal positivity for *S.* Typhi/Paratyphi was available for 19 (46.3%) cases and 15 (35.7%) cases had histopathologic confirmation.

The CFR due to enteric fever ileal perforation was 7.1% in this study, which is less than the 15.4% reported in a systematic review for typhoid ileal perforations in developing countries [8] and 10.5% reported from India [28]. These estimates rely on hospital-based data from select centers in these countries and may not necessarily be representative. While the Global Burden of Diseases (GBD) classifies typhoid/paratyphoid fever as an enteric infection with separate estimates for each pathogen, the National Burden Estimates of India classifies it under the wider umbrella of diarrheal diseases. Hence, a direct comparison of our fatality rates is difficult. The GBD 2017 estimates for age-standardized death rate for enteric fever is 1.9 per 100 000 with 135 900 deaths [29]. In the Million Deaths study conducted in India, about 4.9% of deaths in the 5–14 years age group was due to typhoid [30]. The overall case fatality for hospitalized enteric fever cases is less than 1% when appropriate treatment is instituted. Ileal perforations, on the other hand, often present late and are thus associated with significant mortality. Also, because ileal perforation is a complication that can result in death, a significant proportion of the deaths due to enteric fever could be attributed to perforations.

This study was embedded in a large survey for typhoid in India and hence captured data in facilities that were actively recording and testing for typhoid with standardized approaches. However, the protocol for ileal perforation surveillance did not mandate a blood culture because blood cultures were not expected to have a high rate of positivity, given the late manifestation of ileal perforation in enteric fever [25, 26]. Newer antibody assays such as those targeting HlyE and immunohistochemistry of tissue samples may be useful [31]. Also, being a hospital-based study, the undiagnosed cases and those who did not seek care owing to inaccessibility to health care facilities, especially in rural areas, would have been missed. According to the Government of India’s Medical Certification of Cause of Death report 2017, about 0.2% of the total number of medically certified deaths in India were due to peritonitis not attributed to any specific etiology [32]. It is likely that a significant number among these would have been due to complicated ileal perforations. In the absence of a definite diagnosis of enteric fever, it is possible other conditions may be responsible for ileal perforations, especially in the younger age group due to the challenges in confirmation. Also, the use of Widal test for laboratory confirmation in few of the cases could be one of the limitations of the study.

Overall, the study confirms that enteric fever continues to be the most common cause for nontraumatic ileal perforation in India, followed by tuberculosis, particularly in children. The data are likely to underestimate enteric fever owing to the lack of robust diagnostics and due to the lack of laboratory markers during the perforation phase, which is a late complication.

Given the rise in antibiotic resistance and the emergence of extensively drug-resistant strains of *S*. Typhi in neighboring countries [33, 34], urgent consideration of better prevention measures, such as vaccines, is essential. Not only in the neighboring countries of Pakistan and Nepal, but also in African countries like Malawi and Sierra Leone, where the incidence of typhoid fever has been high for a long time, similar issues obstruct the success of enteric fever control. The World Health Organization prequalified the typhoid conjugate vaccine (TCV) in 2018 for use in endemic countries to prevent typhoid, and hence the risk of emergence of drug resistant strains and late complications like ileal perforation [35]. Malawi is the first African country to host an efficacy trial for the vaccine and it is expected that they would the first to introduce TCV as part of their routine immunization program [36]. Because the burden of ileal perforation, which is a late complication of inadequately treated typhoid, remains substantially high, programmatic TCV introduction needs to be considered seriously by the Indian government.

## Supplementary Data

Supplementary materials are available at *The Journal of Infectious Diseases* online. Consisting of data provided by the authors to benefit the reader, the posted materials are not copyedited and are the sole responsibility of the authors, so questions or comments should be addressed to the corresponding author.

jiab258_suppl_Supplementary_Table_1

## Notes

***Acknowledgments.*** We acknowledge with gratitude the participants and their families for their partnership over the 2 years of surveillance. We also acknowledge the contributions made by central co-ordination team members Arun S Karthikeyan, Ramanujam Karthikeyan, Dilesh Kumar A, and Reshma Raju; and members of the Surveillance for Enteric Fever in India (SEFI) consortium Anna P. Alexander, Philip Alexander, Christina Dhas Sankhro, Dennis Martin, and Roshine Mary Koshy; in addition we acknowledge Jason Andrews, Nathan Lo, Gordon Dougan, Robert Breiman, Megan Carey, and Thomas Cherian for their contribution to the design and analytic plans. Supriya Kumar, Duncan Steele, and Anita Zaidi of the Bill and Melinda Gates Foundation have been steadfast in their support of the study with the constructive comments and encouragement. Our gratitude is also due to Soumya Swaminathan, former Secretary, Department of Health Research, Government of India for providing leadership and participation from the Government in the design and conduct of the surveillance.

***Author contributions.*** S. K. N., G. K., and J. J. conceived and designed the study and developed the study protocol and analysis plan. J. J. and G. K. coordinated the project. S. K. N., M. T., A. K., K. S., P. R., S. A., S. N., J. S., S. P. P., D. R. J., S. T., V. I., P. Z., A. S., M. G., S. E. E., M. S. T., D. G., K. K., M. S., and S. B. coordinated data collection at the sites. S. K. N., M. T., and J. J. analyzed the data. S. K. N. and M. T. drafted the manuscript. All authors have reviewed the manuscript and approved it. J. J. and G. K. had complete access to data and guarantee the manuscript.

***Financial support.*** This work was supported by the Bill and Melinda Gates Foundation (grant number INV-009497-OPP1159351) as part of the National Surveillance System for Enteric Fever in India.

***Supplement sponsorship.*** This supplement is sponsored by the Christian Medical College Vellore Association.

***Potential conflicts of interest.*** All authors: No reported conflicts of interest. All authors have submitted the ICMJE Form for Disclosure of Potential Conflicts of Interest. Conflicts that the editors consider relevant to the content of the manuscript have been disclosed.
