# Supplementary material for: Ileal Perforation and Enteric Fever: Implications for Burden of Disease Estimation
Source: J Infect Dis. 2021 Nov 23;224(Suppl 5):S522–8. doi: 10.1093/infdis/jiab258 (PMC8914860; doi:10.1093/infdis/jiab258)
Supplement: jiab258_suppl_Supplementary_Table_1 [file jiab258_suppl_Supplementary_Table_1.docx]

Table: List of sites in the surveillance

| **S no** | **Site Name** | **Type** |
| --- | --- | --- |
|  | All India Institute of Medical Sciences (AIIMS), Delhi | Urban Tertiary care |
|  | Post Graduate Institute of Medical and Educational Research (PGIMER), Chandigarh | Urban Tertiary care |
|  | Christian Medical College, Ludhiana | Urban Tertiary care |
|  | Topiwala National Medical College- BYL Nair Hospital and Kasturba Hospital, Mumbai | Urban Tertiary care |
|  | St Johns Medical College, Bengaluru | Urban Tertiary care |
|  | Christian Medical College, Vellore | Urban Tertiary care |
|  | Kanchi Kamakoti CHILDS Trust Hospital (KKCTH), Chennai | Urban Tertiary care (Paediatric) |
|  | Chacha Nehru Bal Chikitsalaya (CNBC), Delhi | Urban Tertiary care (Paediatric) |
|  | Lady Willingdon Hospital, Manali | Rural Secondary care |
|  | Sector 45 & 16 Hospitals, Chandigarh | Urban Secondary care |
|  | Makunda Christian Leprosy & General Hospital, Karimganj | Rural Secondary care |
|  | Duncan Hospital, Raxaul | Rural Secondary care |
|  | Chinchpada Christian Hospital, Gangapur | Rural Secondary care |
|  | Rural Development Trust Hospital, Bathalappalli | Rural Secondary care |
